# Balancing Acute and Chronic Occupational Risks: The Use of Nitrile Butadiene Rubber Undergloves by Firefighters to Reduce Exposure to Toxic Contaminants

**DOI:** 10.3390/toxics11060534

**Published:** 2023-06-15

**Authors:** Stijn Everaert, Greet Schoeters, Karel Claes, Jean-Marie Raquez, Bart Buffel, Tamara Vanhaecke, Jonas Moens, Juha Laitinen, Nicolas Van Larebeke, Lode Godderis

**Affiliations:** 1 Chemical Environmental Factors Group, Superior Health Council, 1060 Brussels, Belgium; 2 Department of Biomedical Sciences, University of Antwerp, 2650 Antwerp, Belgium; greet.schoeters@uantwerpen.be; 3 Burn Center & Department of Plastic Surgery, Ghent University Hospital, 9000 Ghent, Belgium; karel.claes@uzgent.be; 4 Polymer and Composite Materials Department, University of Mons, 7000 Mons, Belgium; jean-marie.raquez@umons.ac.be; 5 Department of Materials Engineering, KU Leuven, 8200 Bruges, Belgium; bart.buffel@kuleuven.be; 6 Department of In Vitro Toxicology and Dermato-Cosmetology, Vrije Universiteit Brussel, 1050 Brussels, Belgium; tamara.vanhaecke@vub.be; 7 Belgian Poison Centre, 1120 Brussels, Belgium; jonas.moens@poisoncentre.be; 8 Pelastusopisto, Emergency Services Academy Finland, 70821 Kuopio, Finland; juha.laitinen@pelastusopisto.fi; 9 Department of Radiotherapy and Experimental Cancerology, Ghent University, 9000 Ghent, Belgium; nicolas.vanlarebeke@ugent.be; 10 Department of Analytical, Environmental and Geo-Chemistry, Vrije Universiteit Brussel, 1050 Brussels, Belgium; 11 Center for Environment and Health, Department of Public Health and Primary Care, KU Leuven, 3000 Leuven, Belgium; lode.godderis@kuleuven.be; 12 IDEWE, External Service for Prevention and Protection at Work, 3001 Heverlee, Belgium

**Keywords:** nitrile butadiene rubber, undergloves, burn, exposure, risk assessment, firefighters

## Abstract

Firefighters are exposed via multi-route exposure to a multitude of chemicals (PAHs, VOCs, flame retardants, dioxins, etc.) that may cause acute and long-term health effects. The dermal absorption of contaminants is a major contributor to the overall exposure and can be reduced by wearing appropriate personal protective equipment. As leather firefighters’ gloves cannot be decontaminated regularly by wet cleaning, many Belgian firefighters wear supplementary undergloves made of nitrile butadiene rubber (NBR) to protect against the accumulation of toxicants. However, the safety of this practice has been questioned. In this commentary, the current practice and risks are outlined for the first time, assessed by an interdisciplinary working group of the Belgian Superior Health Council. As NBR sticks to the skin more at high temperatures, the contact time on removal will be prolonged, posing an additional risk for deeper burns. However, based on the physicochemical properties of NBR and the existing experience of firefighters and burn centers, it is estimated that such incidents occur relatively rarely in practice. On the other hand, the risk of repeated exposure to contaminated gloves if no undergloves are worn is unacceptable. Despite the slightly increased risk for deeper burns, it is concluded that wearing disposable NBR gloves under regular firefighters’ gloves is an appropriate and effective preventive measure against toxic contamination. The nitrile butadiene rubber must always be fully covered to avoid any contact with the heat.

## 1. Introduction

**Figure 1:**
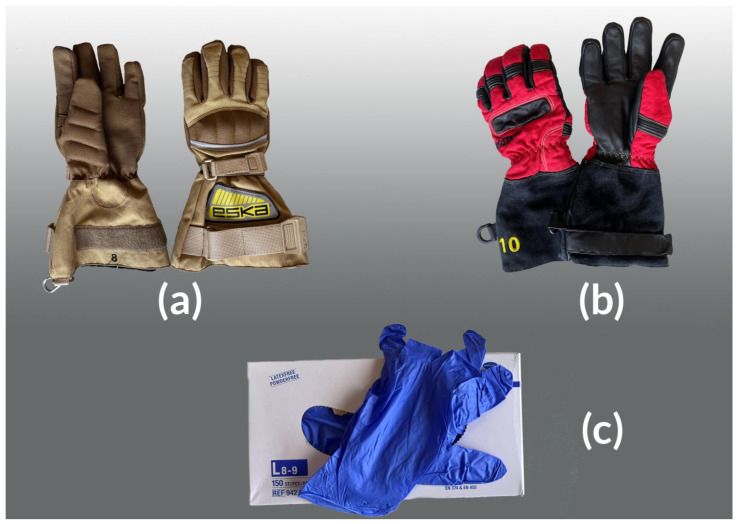
Different types of gloves used by Belgian firefighters: (**a**) textile firefighters’ gloves; (**b**) leather firefighters’ gloves; (**c**) disposable undergloves consisting of nitrile butadiene rubber. Pictures provided by F. Bodeux (Verviers firefighters).

During interventions, firefighters are exposed to a diverse cocktail of carcinogenic substances [1,2,3,4,5,6,7,8]. The International Agency for Research on Cancer (IARC) recently announced a reclassification of the occupational exposure of firefighters from “possibly carcinogenic to humans” (Group 2B) to “carcinogenic to humans” (Group 1) [9]. Due to the widespread use of highly effective self-contained breathing apparatus (SCBA), dermal absorption is now considered to be the major route of exposure [2,3,4,5,6]. Therefore, proper use of qualitative personal protective equipment (PPE) is paramount to protect firefighters against both toxic exposure (chronic risks, e.g., cancer) and physical harm (acute risks, e.g., burns). Protection of the hands deserves specific attention, as they are constantly used during interventions to manipulate different tools. Afterwards, contaminated hands might increase firefighters’ gastrointestinal tract exposure through contact with the mouth. All firefighting gloves are certified according to the European Standard EN 659, but only few reports testing their protection efficiency have been published. In a recent British study, firefighters’ potential exposure to PAHs was measured on the surface of the gloves, while the actual exposure was measured on the surface of their hands [5]. The protection level of the gloves showed large variations, ranging from almost no protection to an exposure reduction of about 90%. In general, structural firefighters’ gloves consist of three layers: an outer shell (textile or leather), a moisture barrier (breathable, waterproof membrane) and a thermal barrier/lining (aramid fibers, e.g., Kevlar). Textile firefighters’ gloves are very suitable as they can be decontaminated relatively easily by means of appropriate washing protocols. However, some Belgian firefighters prefer the use of leather firefighters’ gloves rather than textile gloves (Figure 1), because the former are perceived to be more comfortable against heat. Nevertheless, leather gloves cannot be decontaminated as effectively because they degrade faster by repeated (wet) cleaning. To protect the skin from the possible accumulation of harmful contaminants in the leather gloves, they are often combined with disposable nitrile butadiene rubber (NBR) undergloves [10]. These were chosen by firefighters because of their high impermeability to aqueous solutions and relative resistance to a multitude of chemicals [11,12,13,14,15,16,17,18]. Due to these protective properties against cross-contamination, NBR gloves are also worn by researchers when collecting wipe samples from contaminated skin surfaces and PPE after fire interventions/exercises [2,3,19]. However, concerns were raised by policy makers whether the use of NBR undergloves during interventions might increase the risk and severity of burns. This led to an investigation by the Belgian Superior Health Council addressing the justification of the use of NBR undergloves as a protective measure [20]. The objectives of this commentary are (1) to report on the use of nitrile undergloves in firefighters, a preventive measure not yet reported in the scientific literature; (2) to use this case as an illustration of the difficult balancing of chronic and acute occupational risks; (3) to address the need for more case reports about this practice and the incidental occurrence of burns.

## 2. Methods

In 2022, a multidisciplinary working group was set up by the Belgian Superior Health Council with experts active in the following domains: polymer chemistry, plastic surgery, burn treatment, occupational medicine, toxicology, pharmacy, cancerology and the organization of rescue services. A deontological committee evaluated the risk of conflicts of interest for each expert before participation. Peer-reviewed publications were consulted for each sub-aspect: exposure of firefighters to toxicants and associated health effects; physicochemical properties of NBR; occupational incidence, prevention and treatment of burns. For each subtopic, an extensive literature review was performed using databases such as PubMed, Web of Science and Scopus, search engines such as Google Scholar and relevant references within articles. The strategy and selection criteria varied depending on data availability and subtopic importance. When insufficient scientific literature was available, a conservative expert risk estimate was made by consensus. To include field experience, two independent hearings were organized with representatives of the Belgian fire rescue zones Brussels and Verviers. The position of the working group was adopted unanimously, after which recommendations for further research were identified.

## 3. Results and Discussion

### 3.1. Exposure of Firefighters

#### 3.1.1. General Exposure and Adverse Health Effects

During interventions, firefighters are repeatedly exposed to high concentrations of (geno)toxic, endocrine-disrupting and/or carcinogenic compounds. Important groups are polycyclic aromatic hydrocarbons (PAHs), volatile organic compounds (VOCs), brominated and organophosphate flame retardants, dioxins, furans, phthalates, polychlorinated biphenyls (PCBs), per- and polyfluoroalkyl substances (PFAS) and carcinogenic raw materials such as asbestos [1,2,3,4,5,6,7,8,21,22]. Traditionally, PAHs receive the most attention in studies on firefighters’ exposure [2,6]. However, the diversity of the toxicant cocktail has increased in recent decades, simultaneously with the rise in synthetic materials in construction. An illustration is the high concentration of the plasticizer di-(2-ethylhexyl)phthalate (DEHP) detected on used protective clothing by Alexander and Baxter [23]. Phthalate diesters such as DEHP are found in high concentrations in polyvinyl plastics in wire sheathing, flooring, vinyl siding, etc.

Adverse health effects usually manifest themselves in the long term, most likely amplified by additive, synergistic effects of the various toxic substances to which the body is exposed over time. The mechanisms are still not completely understood. Endocrine-disrupting effects are often subtle and even unnoticed in the short term. For example, a recent human biomonitoring study by Trowbridge et al. [24] found an inverse association between the flame retardant bis(1,3-dichloro-2-propyl)phosphate (BDCPP) and thyroxine (T4) levels in female firefighters. Cancer remains the main adverse long-term effect, as evidenced by several large epidemiological studies [25,26,27]. While the occupational exposure of firefighters was classified before as “possibly carcinogenic to humans” (IARC group 2B) [28], Demers et al. [9] announced that this classification will be updated to “carcinogenic to humans” (IARC group 1) in the upcoming IARC Volume 132. Sufficient evidence was found for a causal association between occupational exposure as a firefighter and the elevated incidence of mesothelioma and bladder cancer. Asbestos is a plausible causal agent for mesothelioma, while PAHs and soot were related to bladder cancer. Limited evidence was found for a causal association with prostate, colon and testicular cancers, besides malignant melanoma and non-Hodgkin lymphoma [9].

#### 3.1.2. Dermal Exposure, Absorption and Adverse Health Effects

Increased cancer risks among firefighters are caused by the total exposure of the body to various carcinogens over a long time. However, this exposure is not homogeneously distributed across all anatomical regions. Variation is caused by differences in the protective efficacy of various PPE and possible incorrect use. Despite being covered by turnout gear and the use of SCBA, Fent et al. [2,4] found significant positive correlations between external exposure and biomarker levels (change in urinary PAH metabolite levels and post-exposure exhaled breath concentrations of benzene). This illustrates how small amounts of toxicants still penetrate through multiple layers of protective clothing and are absorbed by the skin. For the question of firefighter gloves, it is important to quantify the contamination of the hands relative to other body parts.

Fent et al. [2] screened the contamination of different anatomical sites among 15 firefighters for post-exposure PAH levels (six PAHs) using dermal wipe samples and HPLC-PDA. Compared to pre-exposure PAH levels, all post-exposure levels were elevated, but the increase was only statistically significant for the neck (ca. 2% of the total body surface area (TBSA) of an adult male). PAH contamination decreased in the following order: neck > scrotum > face > hand ~ arm. In both rounds, the hands (ca. 5% TBSA) yielded median post-exposure PAH-levels of 15.9 and 23.7 µg m^−2^ compared to 52.0 and 62.8 µg m^−2^ on the neck. In contrast to the findings of Fent et al. [2], Stec et al. [5] found higher concentrations of 12 carcinogenic PAHs (up to 550 µg m^−2^) on the hands of four firefighters, while the concentrations measured on the neck (up to 300 µg m^−2^) and jaw (up to 60 µg m^−2^) were lower. Another strategy was followed by Baum et al. [7] and Levasseur et al. [8], using silicone wristbands worn below PPE. The former study noted that concentrations of low-molecular weight PAHs significantly increased post-intervention. Levasseur et al. [8] detected 134 different chemicals on the wristbands of 20 firefighters, and an occupational association was concluded for PAHs, PFOS, brominated flame retardants and some organophosphate esters. In general, it can be stated that the hands are exposed to a diverse cocktail of toxicants, despite the use of firefighters’ gloves (without the use of undergloves).

The skin absorption of chemical compounds depends on several factors: the physicochemical properties of the substance and its concentration, duration of exposure, temperature, relative humidity, thickness of the skin, sweating, density of sweat ducts and hair follicles. Vanrooij et al. [29] studied PAH absorption for different anatomical sites by applying coal tar ointment. It was estimated that, on average, 20–56% of a low PAH dose was absorbed by the skin after 6 h. After 45 min of exposure, using PAH fluorescence, the average PAH absorption rate constants followed the subsequent ranking: shoulder > forehead, forearm, groin > ankle, hand (palmar site). The interindividual difference was small, while differences between anatomical sites were statistically significant. By contrast, the urinary excretion of 1-OHP (1-hydroxypyrene, a typical PAH metabolite) showed no significant relation to different skin regions. It was estimated that after coal tar ointment application on the skin, 0.3–1.4% of the pyrene dose becomes systemically available. However, it should be noted that the mean excreted 1-OHP concentration was, again, the lowest after application on the hand (7.7 nmol), while the highest concentration was found after application on the neck (14.6 nmol). This may be due to the thicker *stratum corneum* of the epidermis on the palmar sites of the hands, which was also assumed by Stec et al. [5]. As Levasseur et al. [8] showed, there are many other hazardous substances besides PAHs to which firefighters are exposed. Unfortunately, little has been published on the dermal absorption rates of the vast majority of these substances. With regard to VOCs, some studies have used benzene [30,31,32], but these are difficult to interpret because liquid benzene is often used, which does not correspond to exposure during fire interventions. Furthermore, when examining the absorption rates of contaminants by the skin, the moisture should also be taken into account. To ensure the barrier function of the skin, skin hydration needs to be balanced. When the hydration increases, the permeability of the skin may be enhanced manyfold [33]. Increased skin hydration is often seen in occlusive environments, when using PPE or working in a humid environment as firefighters do [34]. In addition, firefighters’ working environments often have high temperatures. Combined with humid conditions, these elevated temperatures can have a synergistic effect, increasing the penetration of chemicals through the skin [34,35,36]. It is also known that chemicals react with the sweat inside PPE (e.g., when dissolved in sweat, hydrogen fluoride becomes hydrofluoric acid), which can also affect the absorption rate of the corresponding chemicals through the skin [37]. In general, independent of environmental conditions, we can assume that the thicker *stratum corneum* will make the skin of the hands less permeable to many substances.

Although the combined exposure contributes to cancer development, it is useful to quantify the share of different anatomical areas. Using cancer slope factors, Stec et al. [5] estimated cancer risk factors for 1 in 100,000 cases per population based on total PAH measurements on the skin and different PPE. Four firefighters were studied before and after a container training exercise. A lifetime risk at age 70 was estimated, with the assumptions of PAH skin absorption being 20% and exposure 2 days/week, 50 weeks/year, for 40 years. A higher risk characterization ratio of 25 cases was calculated based on the concentrations detected on the hands. In comparison, up to 350 firefighters (in a population of 100,000) may develop cancer based on the PAHs found on clothing. Thus, hands have a limited, but not negligible, influence on the total carcinogenic exposure. However, it can be expected that the repeated use of leather gloves without (sufficient) decontamination procedures leads to the accumulation of carcinogens, thereby substantially increasing the actual exposure and associated risks. Therefore, preventive measures are needed.

### 3.2. Experience with Undergloves

The efficacy of undergloves as a preventive measure against contamination was demonstrated by Laitinen et al. [21]. In their study on the exposure of firefighting trainers in smoke diving simulators, the amount of PAHs on the hands decreased by 80% when cotton undergloves were used. During these tests, the instructors did not use extinguishing water and their hands remained dry. At the moment, cotton pimple or leather gloves are used by Finnish instructors as they are perceived to be more comfortable to use during the whole shift. To avoid scald burns, these are only recommended if there is no risk of contact with extinguishing water. In cold smoke trainings and when carrying out maintenance of equipment, NBR gloves are recommended. In France, preventive measures listed by CNRACL [38] mention the use of nitrile or cotton undergloves during the intervention (clearing and surveillance phases) and after the intervention (cleaning of material).

The existing scientific literature on prevention in firefighters has not reported on the use of disposable undergloves made of nitrile butadiene rubber (NBR). However, it is a practice regularly used by firefighters in Wallonia and Brussels (Belgium). In a report of the Liège firefighters, made accessible to our working group, NBR undergloves were explicitly chosen over cotton ones. As cotton will absorb sweat and moisture, it was argued that contaminants may pass due to capillary action [10]. In contrast, NBR has good resistance against water [11] as well as many chemicals in aqueous solutions [14,15]. However, the lack of reported experience has led to discussions on this practice with policymakers and unions. Therefore, the working group invited two representatives of fire brigades from Brussels and Verviers. This hearing revealed that the latter were not aware of any cases where NBR undergloves had led to additional burns during interventions in Belgium. The practice was viewed positively by many firefighters; they often use NBR undergloves in combination with any type of regular firefighter’s gloves (Figure 1), to protect against contamination when removing PPE after the intervention.

In Norway (Bergen), one unpublished case is known of a firefighter with severe burn injuries on the hands after using NBR undergloves during a hot smoke training session (Kristoffersen, pers. comm. 2022). Due to a flashover, a sudden rise in temperature made the moisture on his hands boil. However, the contribution of the NBR undergloves to the burns remained uncertain because the firefighter had not taken appropriate measures in time to cool the temperature of the room.

### 3.3. Properties of Nitrile Butadiene Rubber Gloves

Nitrile butadiene rubber (CAS 9003-18-3) is an elastomer derived from the copolymerization of butadiene and acrylonitrile [11,39]. Variations in its (thermos)physical properties are mainly determined by its acrylonitrile content, varying in single-use NBR gloves between 12.7 and 29.9% [14]. For example, the latter authors reported that the normalized breakthrough detection time for an aqueous solution of captan (phthalimide-class fungicide) increased by 120 min for every 5% increase in acrylonitrile. Additionally, other factors have an influence: increases in glove thickness and area density are associated with increases in breakthrough time and decreases in the steady-state permeation rate [18]. In general, NBR is highly resistant to a multitude of hydrophobic and hydrophilic substances and is, therefore, widely used in both industrial and medical applications [11,12,13,14,15,16,17,18,39,40]. NBR is an elastomeric material and, therefore, cannot melt. The polymer chains of the elastomer are irreversibly cross-linked during curing (vulcanization) and form a covalently bonded molecular network. According to multiple (commercial) material datasheets, NBR remains stable in a broad range of operating temperatures, with minima between −35 and −25 °C and maxima between 100 and 120 °C. Above the maximal operating temperature, degradation starts. NBR becomes “tacky” and NBR gloves will gradually be stickier to the skin. At very high temperatures >200 °C, thermal combustion or decomposition will take place, emitting very toxic compounds such as hydrogen cyanide.

Wide variation exists in the composition and thickness of NBR gloves between different manufacturers [18], adapted to different uses. Currently, there are no fixed selection criteria for disposable NBR gloves used by firefighters. Glove selection should be based on product-specific chemical permeation data [17]. To avoid tearing, a minimum thickness of 0.12 mm was recommended by the Liège firefighters [10]. In Brussels, NBR gloves of the Ansell TouchNTuff 93-250 type are currently used (P. Bécret, pers. comm. 2023). The palm and finger thicknesses are 0.13 mm (5.1 mil) and 0.20 mm (7.9 mil), respectively [41]. These gloves for single use are certified under EN ISO 374-1:2016 Type B (protection against chemicals; breakthrough time ≥ 30 min against n-Heptane, 40% NaOH, 30% H_2_O_2_ and 37% formaldehyde) and EN 374-5 Virus (protection against bacteria, fungi and viruses). The acceptable quality level is 1.5 [41].

### 3.4. Incidence of Burns on the Hands

Based on the annual average of fireground burn injuries reported to the US municipal fire departments between 2007 and 2011 (*n* = 3970), the head (38%) and arms/hands (30%) were most burned, followed by the neck or shoulders (16%) and legs/feet (8%) [42]. These figures remained fairly constant over the years, but unfortunately, no more recent data have been published by the NFPA. Rabbitts et al. [43] studied outpatient firefighter burn injuries over three years in a burn center in New York (*n* = 164). The head (33.2%) and legs (24.7%) were burned most, while the hands accounted for 9.4%. In contrast, inpatient treatment for more severe burn injuries was predominantly needed for the lower extremities, followed by the head/neck. According to Kahn et al. [44], scald burns of the head, neck, wrists and hands are the most common etiology, occurring when steam or liquids enter the turnout gear through small openings between the sleeves and gloves or the interface of the mask and face. Therefore, these authors concluded that burn injuries often occur in a predictable pattern that has to be mitigated with specific prevention efforts. Until now, no statistics have been available for Belgium. Based on the experiences shared in the working group, firefighters with burn injuries of the hands are rarely seen in Belgian burn centers and university hospitals. Severe burns were mainly observed in the neck region. This is only partly in line with the findings from the literature.

### 3.5. Risk Assessment

Given the large amount of contaminants released from fires and the associated cancer risks [1,2,3,4,5,6,7,8,21,22], it is important to protect the hands from contaminants. Undergloves can be an effective tool for this purpose [21]. After the intervention, no avoidable contact between the skin and soot can be allowed. Therefore, it is important that disposable NBR gloves are worn when removing and cleaning PPE and cold intervention materials. In contrast, during and after interventions, direct contact between nitrile butadiene rubber and potentially hot surfaces cannot be allowed. In this case, firefighting gloves complying with the European standard (EN 659) should always be worn over the NBR undergloves. Although NBR is an elastomeric material and cannot melt, NBR undergloves will become stickier to the skin at higher temperatures. Therefore, as the contact time is proportional to the depth of burns, it can be expected that thermal burn injuries will be exacerbated. However, this supplementary risk should also be put into perspective, as NBR remains relatively stable up to 100–120 °C. Such elevated temperatures will also lead to severe burns without the use of NBR undergloves [45,46]. Based on the limited reported experience, it can be assumed that the increased risk of deeper burns from the NBR undergloves is acceptable, compared to the chronic risk of repeated exposure to carcinogenic contaminants in leather gloves without undergloves. Moreover, if these leather gloves cannot be adequately cleaned, it would be preferable to replace them with more launderable textile gloves, or to use new decontamination techniques such as liquid CO_2_. When regular firefighters’ gloves are combined with NBR undergloves, it is of utmost importance that the latter remain completely covered by the former during the entire intervention. Moreover, it should be ensured that there are no small entrances through which hot extinguishing water or steam can enter the glove.

## 4. Conclusions

The use of NBR undergloves among firefighters is a good example of an occupational health issue where acute and chronic risks have to be weighed up on the basis of incomplete data. The following conclusions can be drawn from this risk assessment:

- Despite being covered by firefighters’ gloves, a small proportion of contaminants still penetrates PPE and reaches the skin. Moreover, if gloves are not decontaminated frequently, contaminants will accumulate. Repeated contact between contaminated (leather) gloves and the skin is not acceptable, as the pores of the skin will open due to sweating. Undergloves may provide highly effective additional protection, as demonstrated by Laitinen et al. [21].
- Although NBR undergloves might pose a supplementary risk for deeper burns due to an increased contact time, such incidents are estimated to occur rarely. Based on the physicochemical properties of nitrile butadiene rubber and existing experience among firefighters and burn centers, this risk is currently estimated to be acceptable.
- As this practice has not been previously addressed in the scientific literature, it is of utmost importance that case studies are reported on burn injuries in firefighters using NBR undergloves. Based on these data, the current estimate should be re-evaluated in the future without any reservations.

## Data Availability

Not applicable. No new data were created in this study.
